# The Elderly Perceived Meanings and Values of Virtual Reality Leisure Activities: A Means-End Chain Approach

**DOI:** 10.3390/ijerph15040663

**Published:** 2018-04-03

**Authors:** Cheng-Shih Lin, Mei-Yuan Jeng, Tsu-Ming Yeh

**Affiliations:** 1 Department of Business Administration, National Quemoy University, Kinmen 892, Taiwan; cyrano@nqu.edu.tw; 2 Department of Leisure Recreation and Management Da-Yeh University, Changhua 515, Taiwan; mei521113@yahoo.com.tw; 3 Department of Industrial Engineering and Management, National Quemoy University, Kinmen 892, Taiwan

**Keywords:** hierarchical value map, leisure activities, means-end chain, personal values, virtual reality

## Abstract

This study uses means-end chain (MEC) techniques to examine the awareness, decision-making procedure, and personal values of the elderly with regard to virtual reality leisure activities. The results of the study show that elderly respondents value virtual reality leisure activities that are fun, safe, and easy. In terms of outcome benefits, elderly respondents value feeling physically and mentally healthy, firsthand experience, and satisfied curiosity. In value terms, elderly respondents hope that their chosen virtual reality leisure activities improve not only their relationships with others, but also their enjoyment, quality of life, and sense of belonging. The results show that, while consumers with different awarenesses of virtual reality leisure activities have different decision-making processes, they share creating “good memories” as the terminal value with the most significant effect. This presents a potential opportunity to promote virtual reality leisure activities. Relevant bodies or enterprises can seek to create good memories in consumers by developing activities that are safe and fun, promote good health, and provide good service, thereby attracting the interest of elderly consumers.

## 1. Introduction

According to the World Health Organization (WHO), the proportion of elderly people over 60 years of age will increase from 11% of the world’s population in 2006 to 22% in 2050. As an aging population is now a global trend, the role of leisure activities in enabling the elderly to enjoy good health and high life satisfaction has become a critical issue. Regular physical exercise has a significant effect on the prevention of disease [1]. However, some studies have shown that most leisure activities undertaken by the elderly are passive; watching television is the primary activity. In the long term, a passive lifestyle can cause cardiovascular disease and degradation of body functions [2]. For elderly people to have a healthy quality of life, it is essential to promote physical activity. However, the physiological changes associated with aging have a psychological impact on the willingness of the elderly to participate in leisure activities. Pender [3] pointed out that the elderly participate in physical activities for the benefits and well-being derived from the activities, and to prevent falls. For the elderly, and especially those with disabilities, it is much easier to get started with virtual reality leisure activities than real-world leisure activities such as tennis, bowling or golf [4]. Aside from their high level of accessibility, virtual reality leisure activities also provide a safe and novel way to have fun that is not limited by the weather or a lack of activity partners. These activities enable participants to easily record their performance and progress, enabling individuals to pursue their own goals. As well as encouraging continued participation through these advantages, virtual reality leisure activities are also an effective way to get physical exercise, aiding the promotion of physical health among the elderly.

However, the elderly are often not willing to accept new technology, sometimes rejecting it completely through a fear of not being able to use it. In view of this, it is necessary to understand the decision-making processes of the elderly with regard to participating in virtual reality leisure activities. However, as the psychology of consumer decision making is extremely complex, positioning is an important strategy in the decision-making process for attracting the attention of consumers. The objective of positioning is to establish the product image in the minds of consumers and increase their willingness to continue to participate. Meaningful positioning can also simplify the decision-making process for consumers [5].

At present, most research on participation in virtual reality activities among the elderly is focused on weight loss through exercise and health promotion [6]. Most studies use an experimental method to compare the consequences of activity participation between an experimental group and a control group [7,8,9]. There is a corresponding lack of research on the overall decision-making process and cognitive structure of participants. Some scholars in the field of leisure studies have called for the use of more qualitative research methods, particularly when looking at the relationship between personal values and behavior. The Means-End Chain (MEC) is a qualitative research technique that stresses the importance of personal values. The MEC links attributes, consequences, and values, and can explain how an individual’s choice of products or services meets consumer expectations. As a result, this approach is increasingly used in the field of leisure studies. The MEC helps us to understand consumers’ decision-making processes [10,11,12].

The research method in the current study combines the MEC with the interview technique of laddering to identify the attributes, outcome benefits, and values of virtual reality leisure activities. Finally, we establish the linkages between the attributes, consequences, and values to understand the potential value orientations of the elderly, enabling relevant organizations to satisfy the needs of the elderly and effectively position their offerings.

## 2. Literature Review

### 2.1. Positioning of Virtual Reality Leisure Activities

Virtual reality refers to an artificial reality, and allows users to enter a virtual world through a computer interface and gain experiences similar to those in the real world [13]. Virtual reality, combined with sound, video, graphics, text, and other technologies, can be an arbitrary operation that involves high interaction and immediate responses, allowing users to have an “Immersed” feeling [14]. In recent years, owing to the rapid development of computer hardware and software, the computer animations generated by virtual reality technology have achieved an almost lifelike level of realism. The somatosensory Wii game console launched by Nintendo in 2006 has achieved worldwide popularity. In particular, the Wii sport virtual reality sports games (e.g., tennis, baseball, bowling, golf, boxing and cyberbike) have transcended traditional video games with their intuitive human-machine interaction and somatosensory operation. Wii sport is simple, safe, and fun, while providing players with the opportunity to exercise, making it a new option for sport, exercise, and weight loss. The Wii has been perceived to be easy to use, to provide a way for the elderly to socialize with others, and to give opportunities to participate in activities in a new way [15]. The sound and light can stimulate the cognitive function of elderly users, while interactive games can promote joint motion. The immediate feedback interface increases self-confidence, and fun gaming experiences help maintain dynamic living. These benefits make virtual reality leisure activities suitable for promotion among elderly people.

However, during the decision-making process, consumers are often faced with an overload of information about products and services. The main way to ensure that products are seen as unique and valuable in the minds of consumers is through positioning [16]. Sengupta [17] proposed that the brand awareness of consumers can be divided into functional benefits and non-functional emotional attributes. In the relationship between the consumer and brands, emotional attributes have received increasing prominence [18] as a core pillar of market differentiation and sustainable competitive advantage [19]. This means that positioning activities are not based on the products themselves, but on customer psychology. Therefore, the positioning of virtual reality leisure activities refers to the formation of perceived value through actual consumer participation that distinguishes the product from other products. If the value of virtual reality leisure activities cannot be transmitted to the consumer through participation, virtual reality products cannot be successful in the market in the long term [20].

### 2.2. Benefits of Virtual Reality Leisure Activities for the Elderly

Participation in leisure activities can bring benefits [21]. For the elderly, leisure activities play an important role in social interaction, health promotion, health care, and the prevention of social isolation [22]. Research has shown that leisure activities are important for good health in old age [23] because they can help to improve quality of life for the elderly, their families, and their communities [24], and help them to cope with stress [25]. Therefore, leisure activities play an important role in elderly people’s life satisfaction, mental health, and quality of life.

The demand for leisure activities among the elderly increases as individuals gain more leisure time through retirement or reduced working hours [12]. According to activity theory, the more types of leisure activities elderly people participate in, the greater leisure benefits they can attain [26]. This theory is based on the belief that most elderly people have the ability to maintain a certain level of activity, and that an increase in leisure participation and leisure involvement will enable elderly people to more actively participate in activities, reaffirming their self-worth and increasing life satisfaction [27]. However, owing to the decline in physiological functions and reduced mobility, the elderly have considerably more difficulty with participating in activities than young people do [28]. For the elderly living in cities, rising housing prices caused by overcrowding and an increased demand for land have led to substantial reductions in urban green spaces, meaning that some larger leisure and sports facilities have moved to the edges of cities [29]. As a result, the overcrowded leisure facilities and inconvenient locations have reduced the opportunities for the elderly to participate in leisure activities. In recent years, technological development has enabled the emergence of virtual reality as an alternative leisure activity. Individuals can exercise in their homes, and they are no longer subject to the constraints of the outdoor environment or weather conditions, enabling them to experience the pleasure of leisure activities indoors. Virtual reality technology can introduce different virtual reality or real-life scenarios, enabling people to travel the world from the comfort of their homes, thus integrating physical exercise and leisure tourism. Therefore, when Nintendo launched its new game console, the Wii, the console quickly became a favorite choice of leisure activity for many people. The virtual reality environment provided by the Wii combines high levels of safety with low cost, making it very suitable for leisure use by the elderly. Research by Saposnik et al. [30] has shown that the somatosensory Wii game console is safe, can be used for leisure and fitness activities, and is particularly suitable for the elderly.

In related literature, Farrow and Reid [4] used qualitative research methods to examine the experiences of 16 stroke survivors engaging in sport-based virtual reality leisure activities. It was found that after participating in these activities, the stroke survivors showed a significant increase in flow experiences and leisure competence. Wollersheim et al. [31] also found that the elderly have a relatively higher level of interest in sport-based virtual reality leisure activities than in real-world leisure activities, meaning that they are better able to gain enjoyment through participation in the former. Pigford and Andrews [32] found that when the elderly used the Wii for rehabilitation purposes, they were more willing to engage in rehabilitation and more strongly motivated to participate in the activities; they rarely dropped out. Williams et al. [7] carried out a study of 21 elderly people aged 76 and over in a senior living community. Each respondent played Wii Fit somatosensory games twice a week for 12 weeks. The study found that somatosensory Wii Fit games can improve the balance and self-confidence of the community residents. Further, the acceptance level of somatosensory Wii Fit games among the elderly respondents was high. Examining improvements in balance, Chiang et al. [9] divided 58 elderly respondents into an experimental group and a control group. Experimental activities were conducted on the Wii Sport and Wii Fit three times a week for 45 min each time, for a total of eight weeks. The study found that balance and posture stability were significantly better for the experimental group compared to that of the control group (*p* < 0.05), indicating that these activities can effectively improve balance. Rosenberg et al. [33] tested 19 subjects, with an average age of 78.7 years, who used the Wii Sport for 35 min, three times a week for 12 weeks. Bateni [8] divided 18 elderly participants equally into a traditional physical therapy group, a Wii Fit therapy group, and a combined traditional physical therapy and Wii Fit therapy group. The Wii Fit intervention was divided into two six-week periods, making a total of 12 weeks, with activities occurring three times a week. Berg balance scale testing was carried out before, during, and after the intervention. The results showed that the group that combined traditional physical therapy with Wii Fit therapy achieved the greatest improvements in balance, thus providing empirical evidence for the health promotion benefits of virtual reality leisure activities among elderly people.

### 2.3. Personal Values and Consumer Behavior

Values refer to a belief that one type of behavioral pattern or object is ultimately superior to another type of behavioral pattern or object [34]. Values are important predictor variables in consumer behavior, acting as a firmly rooted set of beliefs and important assessment criteria for individual behavior. How personal values react to different situations explains an individual’s behavioral expression, determining words and actions or how things are evaluated, as well as the level of importance attached to things [34]. Some studies have examined the effect of personal values on the decision-making aspect of human behavior [35]. Both personal internal and external values affect behavior [36]. These differences in behavior increase the function of the cognitive decision-making model [37]. In other words, incorporating personal values enables us to more accurately predict consumer attitudes [38]. According to Peter and Olson [39], value represents desired or useful goals. Individuals may use the ownership of a product or the process of participation to satisfy their individual values. From an experience perspective, Holbrook [40] argued that consumer values are a type of preference, which is influenced by individual likes and interests. Consumer values including self-esteem and affection are used to communicate the attributes of a product or service to the consumer [41]. Therefore, when consumers evaluate products or services, values are an important factor. In other words, behavior is only meaningful when the product features or attributes can satisfy certain basic needs or values. Gutman [42] also asserted that linking consumer behavior and values can strengthen marketing planning and strategy.

### 2.4. Means-End Chain Theory

The purpose of the MEC is to explore how individuals choose products or services to fulfill a desired end state. The MEC gives a theoretical basis for a potential relationship between the consumer decision-making process and cognitive structure. The MEC, proposed by Gutman [42], involves analyzing personal values to reveal the multiple levels of the cognitive structure of decision makers [43]. “Means” refers to the subjective perception of product attributes held by decision makers, while “ends” refers to the values held by individuals. The main purpose of this approach is to understand individual decision making and connect the two nodes to explain consumer behavior [41]. Consumer behavior could be predicted based on how the choices of specific attributes can help individuals realize desired values. Therefore, a three-way relationship exists between “attributes”, “consequences”, and “values” [44].

Using attributes as a method means examining the features of products or services perceived by consumers [45], including concrete and abstract factors such as packaging, price, quality, brand, reputation, and services provided by the sales staff. Consequences refer to direct or indirect effects on consumers after purchasing or using the product. The consequences are explicit outcomes from the purchase, use, or consumption of products, which can be divided into functional and psychological outcomes [39]. Functional outcomes indicate the direct benefits obtained by the consumer following consumption, while psychological outcomes indicate psychological or social outcomes obtained following use. In this context, values, involving consumers’ attempts to obtain important outcomes, indicate psychological behavior, and can be divided into instrumental values and terminal values [39]. Instrumental values indicate behavioral modes adopted to attain the ultimate objectives. Terminal values show consumers’ perceived ultimate ideal state.

The use of MEC has yielded good results in product formulation and advertising strategies [46,47]. As consumers in recent years have increasingly focused on leisure and entertainment consumption decisions, some scholars have started to use MEC in the areas of leisure or virtual products to study the decision-making behavior of consumers [10,48].

### 2.5. Laddering Interviews

Laddering is the earliest method for collecting qualitative data within MEC [49], using one-on-one interview techniques to understand how consumers use product attributes to satisfy their values. The interview process requires an in-depth exploration to uncover the values desired by consumers. The aim of the interviews is to reveal an individual’s motivation for choosing a particular product [50]. Generally speaking, a small or intermediate sample size is used [51].

Laddering interviews can be divided into soft laddering and hard laddering [52,53]. Soft laddering is carried out by applying an open question approach to in-depth one-on-one interviews. This enables respondents to give unrestricted answers, using free elicitation methods to obtain information. Soft laddering analysis requires a sample size of at least 20 [44], but is unsuitable for collecting a large sample [54]. Hard laddering can be carried out using telephone, e-mail, or self-administered questionnaires, collecting information on the hierarchical order of attributes, consequences, and values through the answers from respondents at each level. This approach has a lower cost and is conducive to a large sample size (typically larger than 50) [50,52,54].

Laddering is carried out in three stages. First, respondents are asked to compare and evaluate the major attributes of different products, using the major attributes that are revealed as the starting point for the second stage, where respondents need to explain the relationship between the consequences and their personal values. Interviewers must repeatedly ask respondents, “Why is it important to you?” The level of abstraction is increased in stages (from attributes, consequences, and values) until the respondent is no longer able to answer. The latest stage uses an implication matrix to uncover the association between the attributes, consequences, and values [54,55], depicting the thought process of respondents at different levels as a dendrogram, which is a hierarchical value map (HVM) [56].

In order to prevent the HVM becoming too complex, establishing a cut-off level for the value network is necessary. This requires a decision on the frequency of associations that are included in the HVM. A high cut-off level produces a simple map, with less information and fewer connections, making it easier to explain. By contrast, a low cut-off level produces a complex map containing a lot of information, which is harder to explain [57]. Broadly speaking, a frequency of between three and five is chosen as the cutoff for inclusion in the HVM [56].

## 3. Research Methodology

### 3.1. Research Subjects and Sampling Design

The research subjects were elderly individuals aged 60 and over. Interviews took place over four months between October 2015 and January 2016 in a seniors’ activity center in Taichung, Taiwan. The seniors’ activity center provides social and recreational activities for the elderly. Interviewers travelled to the seniors’ activity center in person to identify the elderly individuals who had previously used the Wii and ask if they were willing to be interviewed. Forty respondents were willing to be interviewed; thus, an interview time was arranged. At a prearranged time, interviewers travelled to the elderly respondents’ activity location to explain the purpose and conduct of the interviews, with two interviewers demonstrating the conduct of the interview. Each interview lasted between approximately 40 and 60 min. In order to avoid influencing the activities of other elderly people at the interview location, one-on-one interviews were held in a quiet room. Interviews started with general questions such as where the respondent was from or what their past experience of playing Wii games was. Respondents were slowly guided into the interview in a relaxed manner. Discussion primarily focused on the reasons for choosing the virtual reality game console, Wii, and identifying linkages between the cognitive attributes of virtual reality leisure activities, outcome benefits, and personal values.

The number of valid samples was 40, including 19 males (48%) and 21 females (52%). In terms of age, the 66–70-year-old group was the largest, with 25 respondents, accounting for 62% of the total. This was followed by the 60–65 age group, with 12 respondents, accounting for 30% of the total. Retirees made up the largest group, with 15 respondents, accounting for 42% of the total. This was followed by homemakers, with six respondents, accounting for 15% of the total. In terms of education, “senior high school and vocational” made up the largest group, with 16 respondents, accounting for 40% of the total. This was followed by “university and college,” with 10 respondents, accounting for 25% of the total. Finally, 32 respondents (80%) lived with family members, while eight respondents (20%) lived alone.

### 3.2. Data Collection

As this is an exploratory study, we used soft laddering as the primary method for data connection and analysis [54]. The interviewers first asked the subjects how they felt about virtual reality games. When the subjects expressed a favorable attitude, we continued the in-depth interview with the following two steps:

Step 1: Elicit attributes by asking the following question: Why do you like playing on the Wii? 

Step 2: Carry out in-depth interviews to explore how the attributes and post-experience outcomes of the virtual reality game console, Wii, are related to personal values.

First, we assessed whether the answers provided during the first step referred to attributes of the virtual reality game console, Wii. We used these attributes as the starting point for in-depth interviews by asking the following questions: Why is this attribute important to you? What benefits or advantages can this attribute bring you? Which values in your own life do you experience through these benefits or advantages? Interviewers systematically guided the respondents’ discussions from attributes to consequences, and then from consequences to personal values. The interview process ended when respondents were unable to continue answering the questions.

### 3.3. Data Analysis

Content analysis on the gathered data was conducted. Analysis was carried out in three steps. First, we used interview transcripts to categorize similar statements. After discussion with three experts (A, B, C) familiar with MEC and research on leisure activities and virtual leisure among the elderly, we excluded unsuitable statements, and named each of the factors, producing 25 factors. For coding, the 25 factors were classified as MEC attributes, consequences, and values (A/C/V) according to factor inter-subjectivity, which determined the operational differences of attributes, consequences, and values. Finally, we carried out a reliability analysis, with the testing as follows:

1. Intercoder Agreement:
  $R=\frac{2M}{N_{1}+N_{2}}$
  *R* = Agreement
  *M* = Frequency of matches between two coders
  *N*_1_ = Coding frequency of first coder
  *N*_2_ = Coding frequency of second coder
2. Composite Reliability:
  $CR=\frac{N\times Average\;inter-judge\;agreement}{1+\left[\left(N-1\right)\times Average\;inter-judge\;agreement\right]}$
  *CR* = Composite reliability
  *N* = Number of coders

We used the Kappa index to calculate the inter-judge agreement between pairs of codes as a measurement index. Kassarjian [58] supposed that having the reliability be greater than 0.85 is satisfactory. In this study, content analysis produced 11 attributes (A), eight consequences (C) and six values (V) (see Table 1). Intercoder agreement for each was 0.91, 0.89, and 0.94, respectively, and reliability was 0.98, meeting the sufficient level recommended by experts.

## 4. Research Results

### 4.1. Description of Attributes, Consequences, and Values

From the content analysis, we obtained seven specific attributes (A) (clear picture, simple operation, ease of use, usefulness, gaming experience, cheap price, and explanation of activities) and four abstract attributes (fun, safe, flexible, and beneficial to health), totaling 11 attributes. From the eight consequences (C), we identified five functional consequences (firsthand experience, learning about virtual reality activities, understanding technology products, health benefits, and increasing emotional connection with family and friends) and three psychological consequences (relaxation, stress relief, satisfying curiosity). From the six values (V), we identified three functional values (enjoyment of life, improved quality of life, and better relationships with others), and three terminal values (good memories, self-gratification, and sense of belonging). In terms of the frequency of responses, of the 11 attributes, fun (17 times) and safe (17 times) were mentioned the most frequently, followed by flexibility, simple operation, and usefulness, in that order. From the eight consequences, relaxation (19 times) and firsthand experience (19 times) were the most frequently cited, followed by understanding technology products, health benefits, stress relief, and satisfying curiosity. From the six values, of the instrumental values, enjoyment of life (15 times) was the most frequently mentioned, followed by better relationships with others, and improved quality of life, in that order. Of the terminal values, good memories (24 times) was the most frequently cited, followed by sense of belonging and self-gratification. Higher frequencies indicate that the factors were more important to consumers.

### 4.2. Implication Matrix

The implication matrix was used to produce the HVM; it is an important tool for integrating the frequencies of association. The chain relationships between attributes, consequences, and values were produced by laddering interviews. The figures within the matrix show the frequency of the direct and indirect linkages between attributes, consequences, and values [45]. Direct linkages indicate no hierarchical relationship, for example, attributes to consequences (A-C) or attributes to values (A-V). Indirect linkages represent a complete hierarchical relationship, for example, attributes to consequences to values (A-C-V). The values before the symbol (;) indicate the frequency of the direct chain links between the factors, while those after the symbol indicate the frequency of the indirect chain links between the factors. The figures indicate the strength of the chain link between the factors. Table 2 shows the implication matrix for the value of the virtual reality leisure activity experiences among the elderly. From Table 2, we found that the 40 respondents constructed a total of 188 value ladders, producing an average of 4.7 ladders for each respondent, with A1-C1-V1 and A5-C5-V3 linked the most frequently.

### 4.3. Hierarchical Value Map

**Figure 1:**
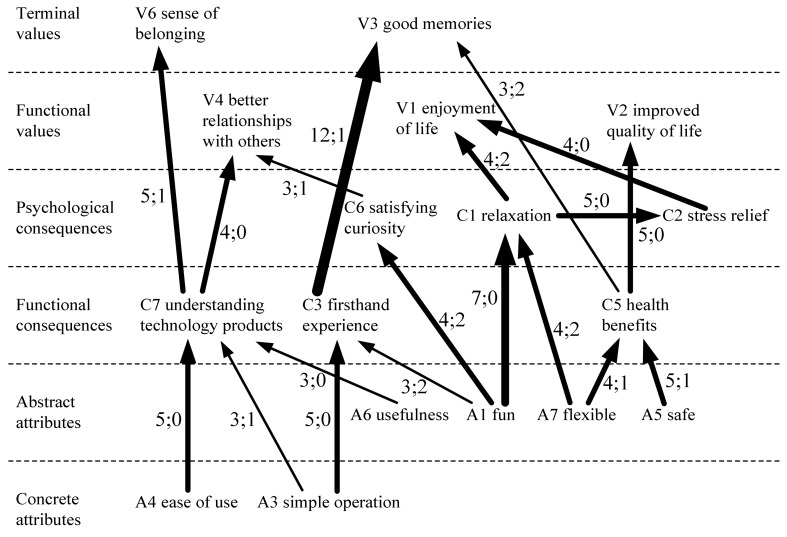
HVM for the virtual reality leisure activity experiences among the elderly (cut-off level is 3).

In order to ensure that the HVM clearly showed the important A/C/V chain relationships, we used Reynolds and Gutman’s [56] suggested cut-off level of three, including A/C/V associations with a frequency of three or more links in the HVM (Figure 1). The frequency of the links between factors is indicated with arrows of differing thicknesses: stronger relationships are shown by thicker lines. Typically, an association with four or fewer links is considered weak, an association with between five and nine links is considered moderate, and an association with 10 or more links is considered strong [59]. The overall analysis showed that the elderly attempted to obtain the functional values of better relationships with others, enjoyment of life, and improved quality of life, and the terminal values of a sense of belonging and good memories. In terms of the strengths of associations, in the frequency of links between attributes and consequences, the association between “fun” and “relaxation” (7; 0) was the strongest, reaching a moderate level, while the highest frequency of links between consequences and values occurred between “firsthand experience” and “good memories” (12; 1), reaching a strong level of association. In terms of the paths of linkages, the consequence of “understanding technology products” was obtained from “ease of use”, “simple operation”, and “usefulness”, which in turn produced the values of “better relationships with others” and “sense of belonging”. Next, “flexibility” and “safety” produced “health benefits” as a consequence, which in turn created the value of “improved quality of life”.

### 4.4. Research Results

The purpose of this study is to use an HVM produced by an MEC and laddering interviews to explore the personal values of elderly respondents and identify the importance of the concrete and abstract attributes of virtual reality leisure activities for elderly respondents. These consequences can be used as a basis for the positioning of virtual reality leisure activities. In terms of the attributes of virtual reality activities, the elderly respondents valued the following six attributes: ease of use, usefulness, simple operation, fun, safe, and flexible. These properties are similar to the technology acceptance model proposed by Davis [60], which identified perceived usefulness and perceived ease of use as the two major cognitive factors. Van der Heijden [61] identified two factors: experiences of fun and perceived usefulness. Thus, it is clear that these attributes are critical to consumers’ acceptance of new technology. 

In terms of outcome benefits, the elderly believed that consumption produced the following five consequences: understanding technology products, firsthand experience, relaxation, stress relief, and health benefits. Of these consequences, firsthand experience was most valued by the elderly respondents, demonstrating the importance of experiences. These results support Pine II and Gilmore’s [62] finding that the four dimensions of entertainment, education, escapism, and aesthetics produce different experiences. In terms of value goals, the elderly respondents hoped to experience improved relationships with others, enjoyment of life, good quality of life, a sense of belonging, and good memories.

## 5. Conclusions

As an aging population is now a global trend, health problems in the elderly create a significant economic burden for the entire national health care system. Participation in leisure activities can delay the negative effects of aging, cultivate a good ability to adapt to aging, and maintain overall healthy living, thus benefitting the physical and mental health of elderly people. The results of this study illustrate the importance of activity participation for elderly people, indicating that the promotion of elderly participation in leisure activities should be strengthened. Regardless of whether these activities are recreational or fitness based, health promotion and the increase of life satisfaction are the most important activity goals.

The question of how virtual reality leisure activities attract the participation of the elderly is a crucial challenge for relevant organizations when promoting activities. Elderly people’s decision-making processes are typically restricted to a given leisure activity. Therefore, organizations must analyze competitors in the market to identify their features and establish a marketing focus. The positioning of virtual reality leisure activities must satisfy consumer needs rather than focus on product functions. 

The findings obtained through this means-end investigation are helpful in understanding the elderly’s underlying values of participation in virtual reality leisure activities. Although qualitative research methods have often been questioned for their rigor, the interview technique of laddering provides a systematic and exploratory strategy, with the interviewer continuously asking questions to allow the elderly respondents to explain more clearly what values they would actually like to obtain through participating in virtual leisure activities. This study provides additional insights into activity attributes and consequences, how they relate to higher order values, and why they are important. They have been described by Rokeach [63] as personally relevant and socially meaningful to the individual and physical activity in the older population. The results from the study show that elderly respondents value fun experiences and safety as activity attributes. Therefore, when organizations and software manufacturers are designing products, they need to be aware of the elderly’s physiological functions and ensure that the pace is not excessive. In terms of the usage space, it is necessary to consider handrails, anti-slip features, interfaces, and text size, and dedicated staff should be on hand to attract and encourage consumers to choose the product. In terms of outcome benefits, elderly respondents value feelings of physical and mental health and relaxation, which are primarily linked to the attribute of “fun experience”. Therefore, it is suggested that fun and relaxing group games are used as a focus for the marketing strategy. In value terms, the Wii provides a user-friendly platform for elderly people to participate in virtual reality leisure activities. The Wii can be played alone (with the other member being the computer system or against the computer system), with two players (e.g., competing or playing against another elderly person), or with multiple players (in the same game, taking turns). The elderly can cooperate with or compete against one another through games, so they can improve both friendship and communication. Elderly respondents choose virtual reality leisure activities in the hopes of achieving better relationships with others, increasing their enjoyment and quality of life, having a sense of belonging, and developing self-actualization. Organizations can provide the opportunity to experience products firsthand, enabling elderly consumers to gain a feeling of accomplishment and meet like-minded friends through participating in activities. Csikszentmihalyi [64] pointed out that happiness is achieved through individual self-cultivation, sufficient preparation, and firsthand experiences. In other words, aside from the benefits of interpersonal relations, elderly people can also obtain self-affirmation and fulfillment through participating in activities, which improve their quality of life and develop a sense of belonging. The results also show that of the terminal values, “good memories” has the highest number of responses (Table 1), and from the frequency of linkages, “good memories” has the strongest linkages (Figure 1). While each respondent may have different views on the attributes, consequences, and values of virtual reality leisure activities, they share the same terminal value of “good memories”. These findings provide a reference for the positioning of virtual reality leisure activities. Organizations can seek to create good memories in consumers by developing activities that are safe and fun, promote good health, and provide good service, thereby attracting the interest of elderly consumers.

This study used a qualitative research method to examine opportunities for the positioning of virtual reality leisure activities based on consumer values. The laddering interviews enabled us to gather generalized data from a small number of respondents. It is suggested that future research uses laddering to conduct in-depth interviews, using the data to create quantitative questionnaires to carry out large-scale surveys, thus generating information from a greater number of samples.

## Figures and Tables

**Table 1:** Definition of Attributes, Consequences, and Values (figures in parentheses indicate the frequency of responses).

Attribute		Consequences		Value	
Specific Attribute	∙ A2 clear picture (4) ∙ A3 simple operation (9) ∙ A4 ease of use (6) ∙ A6 usefulness (8) ∙ A9 gaming experience (3) ∙ A10 cheap price (4) ∙ A11 explanation of activities (3)	Functional Consequences	∙ C3 firsthand experience (19) ∙ C4 learning about virtual reality activities (9) ∙ C7 understanding technology products (15) ∙ C5 health benefits (13) ∙ C8 increasing emotional connection with family and friends (6)	Functional Value	∙ V1 enjoyment of life (15) ∙ V2 improved quality of life (10) ∙ V4 better relationships with others (14)
Abstract Attribute	∙ A1 fun (17) ∙ A5 safe (17) ∙ A7 flexible (10) ∙ A8 beneficial to health (2)	Psychological Consequences	∙ C1 relaxation (19) ∙ C2 stress relief (12) ∙ C6 satisfying curiosity (12)	Terminal Value	∙ V3 good memories (24) ∙ V5 self-gratification (3) ∙ V6 sense of belonging (7)

**Table 2:** Value Implication Matrix for the Virtual Reality Leisure Activity Experiences among the Elderly (*n* = 40).

Type	C1	C2	C3	C4	C5	C6	C7	C8	V1	V2	V3	V4	V5	V6	Total
A1	7; 0	1; 1	3; 2	2; 1		4; 2	0; 2		0; 6	0; 3	0; 5	0; 4			17; 26
A2		1; 1	2; 0		1; 1			0; 1		0; 2	0; 1	0; 1			4; 7
A3		0; 1	5; 0			0; 1	3; 1	1; 0			0; 4	0; 1	0; 1	0; 3	9; 12
A4					1; 0	0; 1	5; 0		0; 2	0; 1	0; 3			0; 1	6; 8
A5	3; 0	2; 0	2; 0	1; 1	5; 1	2; 3		1; 1	0; 3		1; 6	0; 2		0; 1	17; 18
A6			1; 0		1; 0		3; 0	1; 1	1; 1		0; 3	0; 3	1; 0	0; 1	8; 9
A7	4; 2	1; 3		1; 0	4; 1				0; 1	0; 3	0; 2	0; 2	0; 1		10; 15
A8		1; 0							1; 0						2; 0
A9	1; 0		1; 1		0; 1	1; 0			0; 1		0; 1		0; 2		3; 6
A10			1; 0	1; 0		2; 0					0; 1	0; 1			4; 2
A11				1; 0	0; 1	0; 1	2; 0				0; 1			0; 1	3; 4
C1		5; 0	1; 0	1; 0	2; 0	1; 0	1; 0		4; 2	0; 1	1; 3	2; 3	1; 1		19; 10
C2	2; 0		1; 0					1; 0	4; 0	1; 1	1; 1	1; 1	1; 1		12; 4
C3					2; 0	2; 0	1; 0			2; 1	12 ;1	0; 1	0; 1	0; 1	19; 5
C4	1; 0	1; 1			1; 0	1; 0	1; 0		1; 2	0; 1	0; 1	2; 0		1; 1	9; 6
C5	1; 1					2; 0		2; 0		5; 0	3; 2	0; 1			13; 4
C6			1; 0				1; 0		2; 0	2; 1	2; 0	3; 1		1; 0	12; 2
C7						2; 0			2; 0		2; 3	4; 0		5; 1	15; 4
C8	1; 1	1; 0		1; 0							1; 0	2; 1		0; 1	6; 2
Total	20; 4	13; 7	18; 3	8; 2	17; 5	17; 8	17; 3	6; 3	15; 18	10; 14	24; 38	14; 22	3; 7	7; 11	188; 144
